# Metabolic Bone Disorders in Children with Inflammatory Bowel Diseases

**DOI:** 10.3390/life12030423

**Published:** 2022-03-15

**Authors:** Mariusz Olczyk, Elżbieta Czkwianianc, Anna Socha-Banasiak

**Affiliations:** 1 Department of Molecular Pathology and Neuropathology, Medical University of Lodz, Pomorska 251, 92-216 Lodz, Poland; 2 Department of Gastroenterology, Allergology and Pediatrics, Polish Mother’s Memorial Hospital Research Institute, 93-338 Lodz, Poland; elzbieta.czkwianianc@iczmp.edu.pl (E.C.); anna.socha-banasiak@umed.lodz.pl (A.S.-B.)

**Keywords:** IBD, metabolic disorders, pediatrics, bone mineral density, Crohn’s disease, ulcerative colitis

## Abstract

In recent years, there has been a noticeable increase in the incidence of inflammatory bowel diseases in the pediatric population. Entry observations demonstrate anemia, malabsorption, deficiencies in vitamin D and calcium. These aspects, together with the systemic action of pro-inflammatory cytokines and steroid therapy are widely recognized as factors influencing bone metabolism. Presently, however, there are very few studies that can be found in the scientific literature on metabolic disorders in patients with IBD, especially in the pediatric population as the coexistence has not been sufficiently examined and understood. This review aims to summarize the currently available literature, as well as assess which areas have information gaps and need further research.

## 1. Introduction

Inflammatory bowel disease (IBD) is the most frequent chronic digestive tract condition in children. The recently documented significant increased incidence of Crohn’s disease (CD) in the pediatric population [1], including infants, coupled to an ill-defined etiology, is particularly worrisome.

Although clinical presentation, disease evolution, and prognosis of CD in children are similar to CD in adults, pediatric patients have a higher risk of developing extra-intestinal manifestations such as growth retardation [2]. Pediatric patients with IBD are at particular risk for extra-intestinal manifestations of the disease. Entry observations demonstrate anemia, malabsorption, deficiencies in vitamin D and calcium. These aspects, together with the concomitant systemic activity of pro-inflammatory cytokines and steroid therapy, are widely recognized as factors influencing bone metabolism. In the pediatric population, the coexistence of bone metabolic disorders and IBD has not been adequately understood. However, adolescence is a critical period for skeletal development, thus, negligence of symptoms in youth can be a resulting cause of developed consequences during adulthood, even leading to osteoporosis.

This review examines the main factors associated with disturbances of bone metabolism in children with IBD. It also summarizes the current literature and knowledge on bone health in affected patients.

## 2. Background

### 2.1. IBD—Characteristics, Etiology, and Treatment

The IBD group includes Crohn’s disease (CD), ulcerative colitis (UC), but also indeterminate colitis [3]. The CD variant predominates in the pediatric population, accounting for up to 7 out of 10 diagnosed cases and the prevalence is higher in boys than girls for all forms of IBD [4]. These are chronic conditions characterized by periods of remission and exacerbation. Abdominal pain and fever are the more typical symptoms of CD, while UC is manifested mainly by diarrhea. In patients with suspected IBD, an upper gastrointestinal series with small bowel follow-through is often used to detect small bowel involvement. Colonoscopy is preferred over contrast enema, because biopsy specimens can be obtained and visual features can be diagnostic. Findings in UC include diffused carpeting of the distal or entire colon with tiny ulcers and loss of haustral folds. In CD, ulcerations tend to be much larger with a linear, branching, or aphthous appearance. In UC, the colon and rectum are typically affected, where these changes occur continuously. In CD, lesions often occur at the end of the small intestine but can also appear in parts of the large intestine, separated by healthy sections of the intestinal mucosa. Microscopic examination of intestinal biopsies facilitates the final diagnosis. In UC, inflammatory changes are typically confined to the mucosa, whereas segmental and deep inflammation is typical of CD where the inflammation is transmural, with lymphoid aggregates extending to the subserosa [5].

The etiology of these diseases of the gastrointestinal tract is not clear and thoroughly explained. Known facts demonstrate that the disease is determined by genetic and environmental factors with the participation of the patient’s immune system. It is recognized that the occurrence of IBD is influenced by the intestinal bacterial flora, diet, smoking, chemical compounds contained in food and human environments, as well as stress or different medications [6].

Treatment of both main forms is based on anti-inflammatory and immunosuppressive drugs, as well as biological therapy, which in this age group has shown to be particularly effective and safe [7]. Currently, the main goal of treatment in CD is not clinical remission, but the so-called “deep remission” (DR) and an improvement in quality of life which increases with better control of inflammation in the gut.

### 2.2. IBD and Metabolic Bone Disorders—Potential Connections

**Figure 1:**
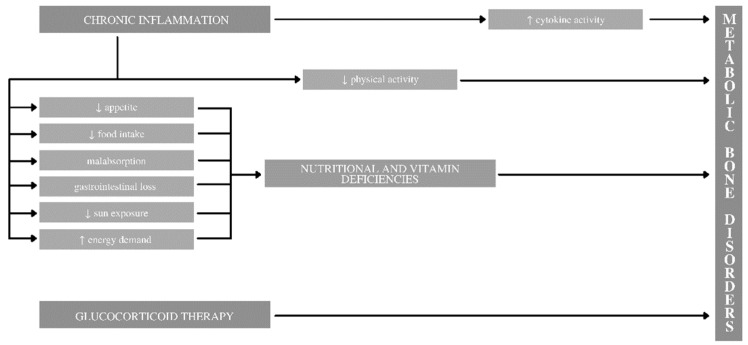
Potential pathways in the course of inflammatory bowel disease leading to metabolic bone disorders in children.

The negative impact IBD causes on bones results due to several systemic mechanisms. In addition to common factors in the pediatric population such as genetic background, age, sex, physical activity, etc., patients with IBD can have a number of additional characteristics, resulting from the effects of intestinal disease. They are presented in Figure 1. 

Figure 1 presents the main causes of metabolic bone disorders resulting from IBD: chronic inflammation, nutritional and vitamin deficiencies, and glucocorticoid therapy. The quality of bone tissue deteriorates in many patients with chronic inflammatory diseases. The basis of these changes is found in immunological processes in which, by disturbing remodeling (including the intensification of resorption or impaired bone formation), it negatively affects the condition of the child’s skeleton. The presence of inflammation leads to the activation of cells of the immune system such as T cells, B cells, macrophages, fibroblast-like synoviocytes, or dendritic cells that secrete cytokines. Unfortunately, some of them may affect the differentiation and activity of osteoclasts and osteoblasts, which consequently may cause deterioration of bone tissue. Consideration should be given to IL-6, TNF-α, and IL-1, which negatively affect bone metabolism [8]. However, studies indicate that there are more cytokines that may affect bone health, such as IL-11, IL-17, IL-18, IL-23, TNF-B, epidermal growth factor (EGF), and prostaglandin E2 (PGE2) [9,10]. Inflammation also indirectly leads to a reduction in physical activity (the impact of which on bone tissue is confirmed by many studies; [11,12,13]), but also loss of appetite, malabsorption disorders, and increased energy demand, which in the case of children and their growing period may be responsible for significant consequences, leading to nutritional and vitamin deficiencies. These deficits, in turn, may already be a direct cause of metabolic bone disorders. The third major cause mentioned in the chart is glucocorticoid therapy, which is also indicated in the literature [14].

Bone metabolic disorders are generalized changes in the structure of the bone tissue resulting from abnormal metabolic processes. This leads to abnormal mineralization of the bone tissue, reduction in its mass, or coexistence of both disorders [15]. The most common form is osteoporosis, in which the processes of bone loss prevail over the processes of bone formation. This is caused by deterioration of the microarchitecture of bone tissue, as a result of which the bone may fracture under relatively light loads [16]. In patients with IBD, the risk of fractures may be increased by approximately 40% [17], due to many factors related to the course of these diseases, unfortunately affecting bone health.

### 2.3. CD and UC—Does One Type Affect Bones More Than the Other?

Most studies in the pediatric population are retrospective and conducted in relatively small groups. As such, there is no agreement as to the effects of different types of IBD on bone metabolism. With that said, an increased predisposition to bone deficits is usually observed with this more common form—CD. Studies have shown that bone metabolism and geometry are altered in pediatric IBD patients; there is a low trabecular mineral density, low cortex thickness, and high cortex mineral density [18]. A recent study shows that no significant differences were found in dual energy X-ray absorptiometry (DXA) results such as bone mineral density (BMD), however, an indicator assessing the microarchitecture of the lumbar spine (the so-called trabecular bone score—TBS) was significantly reduced among pediatric patients with CD, but not among UC patients, which suggests a different effect of these two forms of inflammation on bone microarchitecture [19].

### 2.4. Sex Differences, Growth, Puberty, and Bone Health

The overall frequency of UC cases in Europe is not governed by one gender. However, in the case of CD, some studies indicate more frequent occurrence in women, but these differences are statistically insignificant [20]. Sexual differences have also been observed in the area of bone metabolism. Young men diagnosed with IBD during childhood showed reduced BMD Z-Score values compared to regular standards. It is worth noting that the subjects were statistically shorter, while women diagnosed with IBD during childhood were more prone to delayed puberty compared to the unaffected population [21]. There are, however, discrepancies in the current literature; another analysis showed decreased values of BMD in females, not in males [22], which suggests the need for further research in this area.

In one of the recent prospective studies on bone health in children affected by Crohn’s disease, the scores of densitometric parameters were compared between initial diagnosis and the results obtained at the end of the study. Low bone density at diagnosis was correlated with growth impairment and low BMI [14]. Moreover, BMD values were lower in the lumbar spine than in total body excluding the head, as well at diagnosis or at the end of follow-up [13]. In this study also, almost 20% of patients had a low BMD at the beginning and at the end of the study. Other retrospective studies examining the influence of IBD on growth, puberty, and bone health in children confirm the presence of shorter height, lower BMI, as well as body weight in patients with diagnosed osteopenia based on densitometric parameters [12,23,24,25]. Interestingly, one retrospective longitudinal study on children with IBD showed that improvement in BMD was more pronounced in children who gained weight [26]. These data should be taken into account, especially in the diagnosis of IBD, as a risk factor for bone disease in the pediatric population [12]. Male gender paired with a younger age of onset of the disease may also be associated with an increased risk of linear growth retardation [24].

One recent study found that pediatric patients with IBD frequently continue to grow beyond the expected growth plate closure [27]. Unexpectedly, however, a high number of patients with UC exhibited continued growth, indicating delayed bone age, which is also common in UC. Indication is distinctive of the dynamic marker of disease status, such as growth, and should be monitored regularly into early adulthood, even after patient’s transition from pediatric to adult care.

### 2.5. Chronic Inflammation

Chronic inflammation in the pediatric IBD population is considered to be one of the main factors responsible for low bone mass [28]. The study that examined it, demonstrated that the bone mineral apparent density (BMAD) indicator was used. It applies a transformation of bone area to estimate the volume of each vertebra (L1 to L4) to approximate the effects of bone depth and body size. It is especially recommended for measurements in children and short-stature patients. It has been shown that there is an inverse correlation between BMAD and IL-6 in UC patients [28]. The same publication also draws attention to another important aspect, that is the activity of Crohn’s disease (assessed in the PCDAI scale in children). Similar to IL-6, higher disease activity may predispose to lower bone mineral density [24,28,29].

### 2.6. Treatment Methodology

Biological therapy in children is considered to be a safe and effective method of treatment for both Crohn’s disease and ulcerative colitis [30]. Available evidence demonstrates positive effects and benefits on bone mineral density and bone formation [31]. Infliximab, a monoclonal antibody that binds to the tumor necrosis factor (TNF-α), has been shown to significantly increase bone formation markers such as P1NP (procollagen type 1 N-terminal propeptide) and BSAP (bone specific alkaline phosphatase), consistent with inhibition of TNF-α effects on osteoblasts [32]. Similarly, the increase in resorption markers—serum C-telopeptides (CTX) and deoxypyridinoline (DPD)—reflect the relationship between bone formation and resorption with the therapy [32]. Another prospective study showed that treatment with infliximab in patients with IBD increases the levels of osteocalcin and P1NP. Therapy using this antibody results in concentration of pro-inflammatory cytokines TNF-α, IL-6, a significant decrease in IL-13, as well as improved viability of osteoblasts and their differentiation, giving further evidence of the positive effect of biological therapy on bone metabolism [33].

On the contrary, glucocorticosteroids are often used in the treatment of IBD. However, the study shows that long-term glucocorticoid therapy is considered to be the main clinical risk factor associated with low BMD [14]. One of the aforementioned studies (involving pediatric patients as well as adults with CD) showed that cumulative dose of corticosteroid at the end of follow-up was associated with low BMD [14]. The probable relation is to the reduction in the number and inhibition of osteoblast function. Activation of the glucocorticoid receptor blocks the proliferation of osteoblast precursors, maturation of osteoblasts, and induces their apoptosis [34]. Glucocorticosteroids also affect the function of osteoclasts, but the effect of their function is not sufficiently understood.

Another study comparing pediatric patients with active IBD to patients in remission showed that prior to commencing the glucocorticoid therapy, they were experiencing a reduction in the bone formation markers, as well as lower insulin-like growth factor (IGF-I) along with all bone markers [35]. There was an increase in IGF-I after cessation of glucocorticoid treatment, however, bone formation was impaired as a result of weakness caused by the steroid treatment. It is worth noting that short-term improvements in IGF-1 Z-scores can predict the bone recovery and muscle outcomes following initiation of anti-TNF-α therapy in pediatric CD. These data suggest that the disease causes effects on growth hormone metabolism contributing to musculoskeletal deficits in CD [36].

### 2.7. Physical Activity

There are many publications indicating the positive effect of physical activity on bone density [11,12,13]. In a recent study, a survey was conducted within young adults diagnosed with IBD during childhood. The key was the estimated average number of hours of exercise per week over the past year, where patients were then checked for bone mineral density and body composition. Based on the analysis, it was shown that physically inactive patients had a significantly lower median of total body BMD, skeletal muscle index (SMI, the weight of lean mass in arms and legs/m^2^), and the percentage of fat in Z-scores based on the statistics and compared to patients controls with similar activity. In contrast, highly active IBD patients presented all the above values within the same limits as the control group without diagnosis with corresponding physical exercise levels, although in the former, BMD in the spine and femoral neck were statistically lower [11]. Using multiple regression analyses, a diagnosis of childhood-onset IBD was independently associated with inferior BMD and body composition, regardless of the amount of physical exercise.

One cross-sectional, observational study, conducted in the pediatric IBD population, found a strong positive relationship between moderate-to-vigorous physical activity, lean body mass, and BMD [13]. However, there was no significant correlation determined between daily protein intake and BMD. Another article discussed the possible protective effect of physical activity on the incidence of osteoporosis among IBD groups [12]. Therefore, physical activity—adjusted on an individual basis—should be recommended to IBD patients as an essential element of the behavioral treatment.

### 2.8. Altered Body Composition and Musculoskeletal Deficits

Evidence demonstrates that a diagnosis of IBD during childhood increases the risk of an altered body composition in the future. Myopenic and myopenic-obese body composition profiles were observed much more often in IBD patients than in the control group, and these profiles were strongly associated with low bone density [37]. However, patients with the obese profile showed no significant difference regarding total body BMD Z-score, as compared to patients with a regular profile. On the other hand, research shows that childhood IBD affects bones, not muscles, and bone changes are independent of vitamin D levels [38]. Dynamic muscle function was found to be within the normal reference range, while trabecular BMD and cortical thickness values resulted statistically lower. This could suggest that the disturbance of bone metabolism is a direct consequence of UC or CD disease.

The physiology of muscles and bones is undoubtedly interrelated, therefore, it is worth mentioning the health of muscles in patients with IBD. The so-called skeletal muscle index in CT of the lumbar region is used. Among newly diagnosed patients and previously diagnosed IBD disease, deficits in the Z-score of muscle mass have been found numerously in conducted research [39,40,41,42]. As it turns out, a partial improvement in the results was achieved by effective disease control, while the deficits remained despite the exclusive enteral nutrition and TNF-α therapy [41,42]. In a recent study of low muscle mass in well-controlled young adults with childhood onset CD treated with modern therapies, no abnormal microarchitecture or bone geometry was found in the distal femur, however, muscle deficiencies were found [43]. Unfortunately, these deficits may predispose to future musculoskeletal morbidity, but further research is required in this area.

### 2.9. Vitamin D

Vitamin D deficiency is currently a very common problem which, due to the epidemiological situation in the world, may only worsen. In the pediatric IBD population, according to some studies, up to 62% of patients may not achieve appropriate 25-OHD concentration (>30 ng/mL) [44]. Moreover, vitamin D deficiency is much more common among pediatric IBD patients than in the general population, especially in relation to Crohn’s disease. In one study, only 9.5% of the studied patients with CD had the optimal concentration of 25(OH)D [45]. The same study showed that children with CD or UC were statistically more likely to develop hypocalcemia. Due to the occurrence of malabsorption disorders and increased loss in the gastrointestinal tract, this group of patients has a greater need for supplementation, which, despite its safety and general tolerance, is not always sufficient [46]. However, maintaining the 25-OHD concentration above 30 ng/mL may improve calcium absorption and ensure an adequate range of parathyroid hormone (PTH) [47]. In one of the latest studies, the effect of vitamin D supplementation in children with IBD and its deficiency on disease activity, quality of life, inflammatory markers, and cytokines were examined [48]. The results showed that supplementation significantly decreased IBD activity compared to the placebo group. Furthermore, it has been examined that quality of life also improved in patients with vitamin D supplementation. Inflammatory markers such as erythrocyte sedimentation rate, C-reactive protein, and fecal calprotein and IL-2, -12, -17, -23, TNF-α statistically significantly decreased in the group with vitamin D. Interestingly, the concentration of IL-10 increased after supplementation. Nevertheless, vitamin D supplementation is promising in terms of having a positive effect in children with IBD but requires further research in this area [48].

### 2.10. Other Aspects

**Figure 2:**
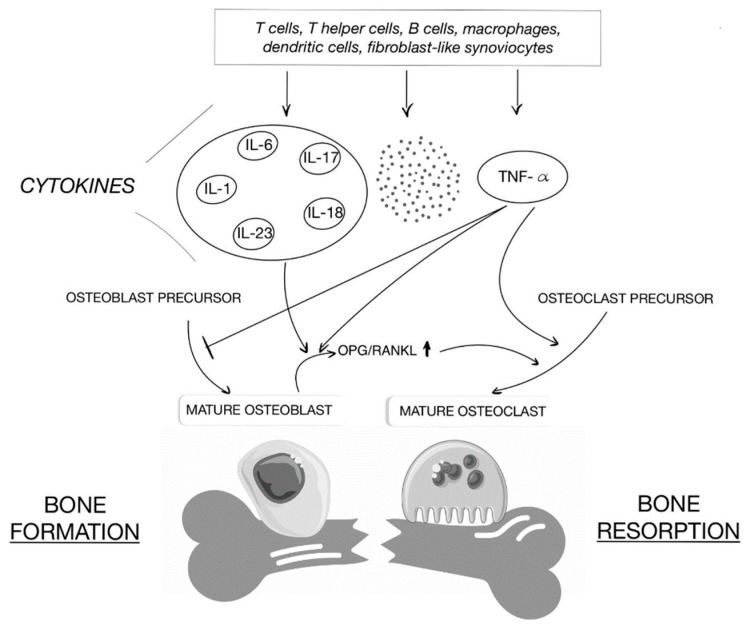
Effects of cytokines on osteoclast and osteoblast formation and bone resorption.

There is growing evidence that osteoimmunology plays an important role in bone metabolism [49]. Focusing on the activator of the NF-κB ligand receptor (RANKL), it plays an important role not only in the development of immune organs and bones, but also in autoimmune diseases affecting bones, such as IBD. The RANKL protein, together with its RANK receptor and its decoy osteoprotegrin (OPG) receptor, regulate osteoclastogenesis. Pro-inflammatory cytokines influence their action, thus creating a link between immunology and bone health. They are presented in Figure 2. 

There are no studies on the incidence of fractures in children with IBD in the available literature. However, in one of the cross-sectional studies involving 80 IBD patients, vertebral fractures occurred in 11% of patients in the study group, compared to 3% in the control group (*p* = 0.02) [50]. Other studies investigating bone health also reported fractures in patients with IBD, but these were isolated cases. [38,39]. Future research is essential in selecting the appropriate methods for assessing bone mass and fracture risk in the pediatric population, as well as the impact of several chronic diseases, such as IBD and others, on the growing and still developing young people [51].

It is claimed that when starting treatment with high doses of glucocorticosteroids in patients, the use of zoledronic acid should be considered, as it effectively prevents the side effects of glucocorticosteroids. The side effects can take the form of loss of bone density, osteoporosis, and fractures. The use of zoledronic acid seems to be the best therapeutic strategy to improve BMD in the lumbar spine [52]. Unfortunately, there are also very few publications pointing to a link between the gut microbiome and bone metabolism. One of the emerging functional benefits of changes in the gut microbiome is increased calcium absorption, increased calcium retention, and improved bone health indicators [53]. Prebiotic fibers increase microbial fermentation in the gut, providing ecological benefits to specific non-pathogenic bacteria that have the ability to modify an individual’s metabolic potential. Fiber fermentation also leads to increased production of short chain fatty acids. These changes were positively correlated with increased calcium absorption in humans and increased bone density and strength in animal models [53]. Dietary fiber may increase calcium absorption with the ability to stimulate the gut microbiome to ultimately affect bone health. However, presently there is a lack of clinical trials in this regard in both children and adults with IBD (Table 1).

## 3. Discussion

Densitometry remains the most widely used tool to detect low bone density. In a child with IBD, a BMD Z-score of less than −2.0 SD is a red light that should lead the patient to a bone specialist to assess if there are other factors that may be contributing to such low bone density scores. The borderline values of the BMD Z-scores, i.e., those between −1.0 and −2.0, especially if they accompany a severe course of the disease, and if there have been numerous fractures of the spinal or long bones in the past, should also be monitored under the watchful eye of a specialist. The most commonly used DXA method is safe, giving high precision of measurements, with minimal exposure to radiation and lasting only a few minutes, which is an important aspect in the case of children. It is recommended to conduct screening and monitoring of BMD in children with certain IBD identified risk factors, control of inflammation with steroid-sparing techniques, nutritional support in children with growth delays and/or lean mass deficits, optimization of vitamin D levels, and weight bearing physical activity [55]. Many IBD patients have abnormalities in the rate of linear growth, usually at diagnosis. It is worth using the height-adjusted Z-score, which takes into account the lower density of smaller bones and allows for a more reliable assessment of BMD [56,57].

Vitamin D supplementation, to achieve and maintain its adequate levels, appears to be particularly important in the treatment of IBD in the pediatric population. It is recommended that these patients have their vitamin D_3_ levels checked at least annually, and even more frequently in patients with additional risk factors, such as severe IBD or glucocorticoid therapy. It seems that supplementation at a dose of 2000 IU/day (in accordance with the latest studies) may reduce disease activity, lower inflammatory markers, and consequently, which is especially important, improve the quality of life of young patients. One cannot forget about the variability of vitamin D concentrations depending on the seasons of the year—the lowest concentrations are recorded at the beginning of the year, and the highest around August [58]. This should be taken into account when comparing vitamin D concentrations in groups of differing patients by factoring in the month of measurement.

**Figure 3:**
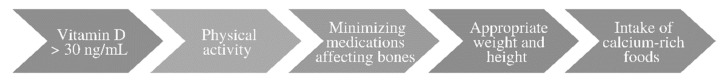
Activities and aspects that require special attention to minimize the risk of bone metabolism disorders in children.

Based on the above analysis, it can be concluded that Crohn’s disease may have a more negative impact on bone health than ulcerative colitis. This is particularly worrisome, as Crohn’s disease predominates in the pediatric population. However, it is difficult to say whether this is due to the pathophysiology of the disease itself, accompanying malnutrition or the method of treatment. The diagram shows the activities and aspects that require special attention to minimize the risk of metabolic bone disorders in children (Figure 3). The figure does not include all the existing factors that increase the risk of bone metabolism disorders, but based on the most important ones, the probability of a negative impact on bone health can be significantly reduced.

Proton pump inhibitors are among the drugs that may affect bone health, not infrequently used in IBD patients, but their link to the risk of fractures in children has not yet been fully elucidated. However, there are already some studies among adult patients indicating a potential relationship between the use of PPIs and fractures [59]. The calcium-rich foods mentioned in Figure 3 refers to the recommended daily intake of 700–1300 mg of calcium per day depending on age [60].

Pediatric IBD patients with an increased risk of musculoskeletal disorders, in addition to classic IBD therapy, should also factor in additional (non-pharmacological) aspects that may affect bones, such as physical activity or adequate nutrition. Further research is needed to determine how these elements should be introduced to best benefit patients with Crohn’s disease or ulcerative colitis.

The pathophysiology of inflammatory bowel diseases with adverse effects on bone health remains unclear. In one of the studies, patients with untreated IBD presented reduced bone turnover, which leads to a higher density of bone matrix mineralization and, consequently, may contribute to the weakening of the general bone strength [54]. Chronic inflammatory diseases through pro-inflammatory cytokines, exposure to glucocorticosteroids, or poor nutrition may disrupt the GH/IGF-1 axis [61]. This is particularly important as the GH/IGF-1 axis is the major regulator of the growth of linear skeleton and muscle accrual during childhood and adolescence [62]. However, future research is essential to having a better understanding of all factors that may affect bone health in children with IBD.

## 4. Conclusions

Inflammatory bowel diseases are becoming increasingly more common in the pediatric population. Metabolic bone disorders at an early stage of development can lead to problems in adulthood. It is important to limit known risk factors for these disorders, as well as conduct and promote further research, as it is crucial to the knowledge that may reveal other important aspects that will allow children to more effectively protect against bone deterioration.

## Figures and Tables

**Table 1:** Summary of scientific findings on metabolic bone disorders in pediatric IBD.

Author (Year)	Design	Study Group Age (Years)	Key Findings	Comments
Sigursdsson et al. (2021) [11]	Cross-sectional cohort	72 IBD, 1341 non-IBD 22.7 (21.3–24.5)	Physical exercise beneficial for BMD	Young adults with childhood-onset IBD
Masip et al. (2021) [12]	Retrospective	57 IBD (27 CD) 11.18 ± 2.24	Suboptimal BMD at diagnosis No difference in BMD between patients with CD and UC ↓ weight and height associated with a ↓ BMD	
Trivić et al. (2022) [13]	Cross-sectional cohort	40 IBD (20 CD, 18 UC, 2 IBD-U) 15.3 ± 0.4	Strong positive relationship between MVPA, LBM and BMD	
Werkstetter et al. (2011) [17]	Prospective cohort	102 IBD (82 CD) New IBD: 30	↓ trabecular BMD, ↓ cortical thickness, and ↑ cortical BMD	Parameters measured by pQCT at the forearm
Levy-Shraga et al. (2021) [18]	Retrospective	50 IBD (35 CD) 13.8 ± 3.0	↓ TBS only in CD	BMD measurements by DXA
Sigurdsson et al. (2017) [20]	Prospective cohort	74 IBD (25 CD) 22.9 ± 2.4	↓ BMD Z-score (lumbar spine and total hip) No difference in BMD between patients with CD and UC	Young adults with childhood-onset IBD
Gokhale et al. (1998) [21]	Prospective cohort	99 IBD	↓ BMD Z-score Cumulative corticosteoroid dose as a predictor of ↓ BMD	BMD measurements by DXA
Rozes et al. (2021) [22]	Retrospective	193 CD	↓ BMD Z-score Long-term glucocorticoid therapy as a predictor of ↓ BMD	
Jin et al. (2021) [23]	Retrospective	127 IBD (117 CD) 13.5 ± 2.5	↓ Vitamin D, ↓ weight-SDS, ↓ IGF-1-SDS, ↓ testosterone, osteoporosis	
Sawczenko et al. [25]	Retrospective	123 CD	↓ Final height in comparison with target height	
Levy-Shraga et al. [26]	Retrospective	41 IBD 12.1 ± 3.5	↑ BMD more pronounced in children who gained weight	Two BMD measurements by DXA, mean interval between the scans 3.4 ± 2.0 years
Gupta et al. [27]	Retrospective	3007 IBD (76% CD)	Growing beyond the time of expected growth plate closure	
Paganelli et al. [28]	Retrospective	56 IBD (35 CD)	Inverse correlation between BMAD and IL-6 in patients with UC Disease activity indexes inversely correlated with BMAD Beneficial effect of IFX on bone density	Cumulative dose of corticosteroids and duration of therapy with no correlation with BMAD
Ronel et al. [29]	Retrospective	116 CD	Osteopenia in nearly half of children with newly onset CD	
Pilcher et al. [31]	Retrospective	33 IBD 13.5 *	After treatment with IFX: ↑ weight, positive catch-up growth, ↑ vitamin D, ↔ bone mass	
Thayu et al. [32]	Multicenter, randomized controlled trial	101 CD 13.3 ± 2.5	IFX therapy associated with ↑ BSAP and ↑ P1NP, inhibition of TNF–α effects on osteoblasts ↑ CTX-1 and ↑ DPD reflect coupling of bone formation and resorption, ↑ linear growth ↑ Risk for having altered body composition traits	
Sigursdsson et al. (2020) [35]	Cross-sectional cohort	94 IBD (29 CD) 18–27 years	Myopenic and myopenic-obese body composition profiles associated with ↓ BMD	Young adults with childhood-onset IBD
Vihinen et al. (2008) [36]	Prospective cohort study	22 IBD 12.3 **	↓ Bone formation in children with active IBD ↓ Bone turnover due to glucocorticoid treatment	
DeBoer et al. (2018) [37]	Prospective cohort study	63 CD	IGF-1 Z-scores predicted recovery of bone and muscle outcomes following initiation of anti-TNF-α therapy	
Maratova et al. (2017) [38]	Prospective cohort study	70 IBD 13.8 *	Altered bone density and geometry but normal dynamic muscle functions	Parameters measured by pQCT
Alkhouri et al. (2013) [39]	Retrospective study	61 IBD (46 CD) 12.3 ± 2.5	↓ Vitamin D	
Ward et al. (2017) [40]	Prospective cohort study	73 CD 7.0–17.7	Profound muscle and bone deficits in children with newly diagnosed CD	Parameters measured by DXA and pQCT
Bechtold et al. (2010) [41]	Cross-sectional study	143 IBD (98 CD) 13.9 ± 3.5	Bone disease in children with IBD seems to be secondary to muscle wasting With longer disease duration, bone adapts to the lower muscle CSA	Parameters measured by pQCT
Werkstetter et al. (2013) [42]	Prospective cohort study	Newly diagnosed CD 10.6–17.7	Disturbed bone remodeling and severely impaired muscle mass in newly diagnosed CD children Bone metabolism and muscle mass improved after starting EEN	
Griffin et al. (2015) [43]	Prospective cohort study	74 CD	Anti-TNF-α therapy associated with ↑ trabecular BMD and ↑ cortical structure	
Steell et al. (2020) [44]	Prospective cohort study	27 CD 23.2 *	Muscle deficits, no abnormal bone microarchitecture or geometry at the distal femur	Young adults with childhood-onset IBD
Jasielska et al. (2021) [46]	Prospective cohort study	74 IBD (43 CD) 14.07 ± 3.58	Low-lactose diet with no effect on BMD	
Amrousy et al. (2021) [48]	Randomized double-blind controlled clinical trial	120 IBD	Vitamin D supplementation decreased the IBD activity score	
Laakso et al. (2012) [51]	Cross-sectional study	80 IBD 14.9 *	↓ BA-adjusted lumbar spine and ↓ whole-body aBMD and ↓ whole-body BMC adjusted for height	
Misof et al. (2017) [54]	Prospective cohort study	20 IBD 14.5 ± 2.3	Children with treatment-naïve IBD: ↓ bone turnover leading to a higher bone matrix mineralization density	

IBD, inflammatory bowel disease; CD, Crohn’s disease; UC, ulcerative colitis; IBD-U, inflammatory bowel disease-unclassified; BM(A)D, bone mineral (apparent) density; MVPA, moderate-to-vigorous physical activity; LBM, lean body mass; pQCT, peripheral quantitative computed tomography; CSA, cross-sectional area; TBS, trabecular bone score; DXA, dual-energy X-ray absorptiometry; IFX, infliximab; SDS, standard deviation scores; IGF, insulin-like growth factor; EEN, exclusive enteral nutrition; BA, bone area; BMC, bone mineral content; ↓, low; ↑, high; ↔ unaffected; *, median age; **, mean age.

## Data Availability

Not applicable.
